# ADAMTS-7 is associated with a high-risk plaque phenotype in human atherosclerosis

**DOI:** 10.1038/s41598-017-03573-4

**Published:** 2017-06-16

**Authors:** Eva Bengtsson, Karin Hultman, Pontus Dunér, Giuseppe Asciutto, Peter Almgren, Marju Orho-Melander, Olle Melander, Jan Nilsson, Anna Hultgårdh-Nilsson, Isabel Gonçalves

**Affiliations:** 1 Experimental Cardiovascular Research Unit, Department of Clinical Sciences, Malmö, Lund University, Malmö, Sweden; 2 Vascular Center Malmö, Skåne University Hospital, Malmö, Sweden; 3 Department of Clinical Sciences, Malmö, Lund University, Malmö, Sweden; 4 Diabetes and Cardiovascular Disease Genetic Epidemiology Unit, Department of Clinical Sciences, Malmö, Lund University, Malmö, Sweden; 5 Department of Internal Medicine, Skåne University Hospital, Malmö, Sweden; 6 Department of Experimental Medical Science, Lund University, Lund, Sweden; 7 Department of Cardiology, Skåne University Hospital, Lund, Sweden; 8 Novo Nordisk A/S, Bagsvaerd, Denmark

## Abstract

Several large-scale genome-wide association studies have identified single-nucleotide polymorphisms in the genomic region of A Disintegrin And Metalloproteinase with ThromboSpondin type 1 repeats (ADAMTS)-7 and associations to coronary artery disease. Experimental studies have provided evidence for a functional role of ADAMTS-7 in both injury-induced vascular neointima formation and development of atherosclerotic lesions. However, whether ADAMTS-7 is associated with a specific plaque phenotype in humans has not been investigated. Carotid plaques (n = 206) from patients with and without cerebrovascular symptoms were analyzed for expression of ADAMTS-7 by immunohistochemistry and correlated to components associated with plaque vulnerability. Plaques from symptomatic patients showed increased levels of ADAMTS-7 compared with lesions from asymptomatic patients. High levels of ADAMTS-7 correlated with high levels of CD68-staining and lipid content, but with low smooth muscle cell and collagen content, which together are characteristics of a vulnerable plaque phenotype. ADAMTS-7 levels above median were associated with increased risk for postoperative cardiovascular events. Our data show that ADAMTS-7 is associated with a vulnerable plaque phenotype in human carotid lesions. These data support previous observations of a potential proatherogenic role of ADAMTS-7.

## Introduction

Several large-scale genome-wide association studies (GWAS) have identified SNPs in the genomic region of A Disintegrin And Metalloproteinase with ThromboSpondin type 1 repeats (ADAMTS)-7 and associations to coronary artery disease (CAD)^1–3^. ADAMTS-7 is a proteolytic enzyme and its best characterized substrate is the cartilage oligomeric protein (COMP) also known as thromobospondin-5^4^. Previous studies have focused on the role of ADAMTS-7 in cartilage degradation, and recently ADAMTS-7 was shown to enhance both osteoarthritis and collagen-induced arthritis in mice^5, 6^. In a rodent model of vascular disease, ADAMTS-7 has been implicated in restenosis^7^, which was recently confirmed in two studies using ADAMTS-7-deficient mice^8, 9^. The role of ADAMTS-7 in neointima formation is mediated via increased smooth muscle cell (SMC) migration by degradation of COMP and via impaired re-endothelialization^7, 9^. In agreement with this, the SNP (identified by GWAS) rs3825807 (A to G), leading to a Ser-to-Pro substitution in the prodomain of ADAMTS-7, reduces cleavage of the prodomain of ADAMTS-7, SMC-mediated COMP degradation, and SMC migration^10^. In addition to its role in restenosis, a recent study showed that ADAMTS-7-deficiency significantly reduces atherosclerotic lesion formation in both ApoE^−/−^ and LDLr^−/−^mice^8^. In cultured SMCs, ADAMTS-7 expression is up-regulated by inflammatory cytokines such as TNF-α, interleukin-1β, reactive oxygen species (H_2_O_2_), and platelet-derived growth factor-BB, whereas the anti-inflammatory cytokine transforming growth factor-β reduces ADAMTS-7 expression^7, 9^. ADAMTS-7 is also expressed in monocytes/macrophages, and interferon-γ or TNF-α stimulation of THP-1 macrophages increases ADAMTS-7 expression in these cells^11^.

Plaque rupture is responsible for the majority of acute coronary events and ischemic strokes^12, 13^. Stable atherosclerotic plaques are characterized by a thick cap of matrix-producing SMCs protecting the plaque from rupture, whereas vulnerable plaques contain a large lipid-filled core covered by a thin fibrous cap, infiltrated by protease-secreting macrophages^14^. Expression of ADAMTS-7 has previously been shown in human carotid atherosclerotic lesions and in human coronary arteries^8, 10^. However, whether ADAMTS-7 is associated with a stable or vulnerable plaque phenotype has not been investigated. In this study we analyzed ADAMTS-7 levels in 206 human carotid plaques and its association with components that affect plaque stability. We found increased ADAMTS-7 levels in lesions from symptomatic patients. Furthermore, ADAMTS-7 levels correlated with plaque components characteristic of a vulnerable plaque phenotype. In addition, high ADAMTS-7 levels were associated with an increased risk for postoperative cardiovascular (CV) events.

## Results

### ADAMTS-7 is associated with a vulnerable plaque phenotype

**Figure 1:**
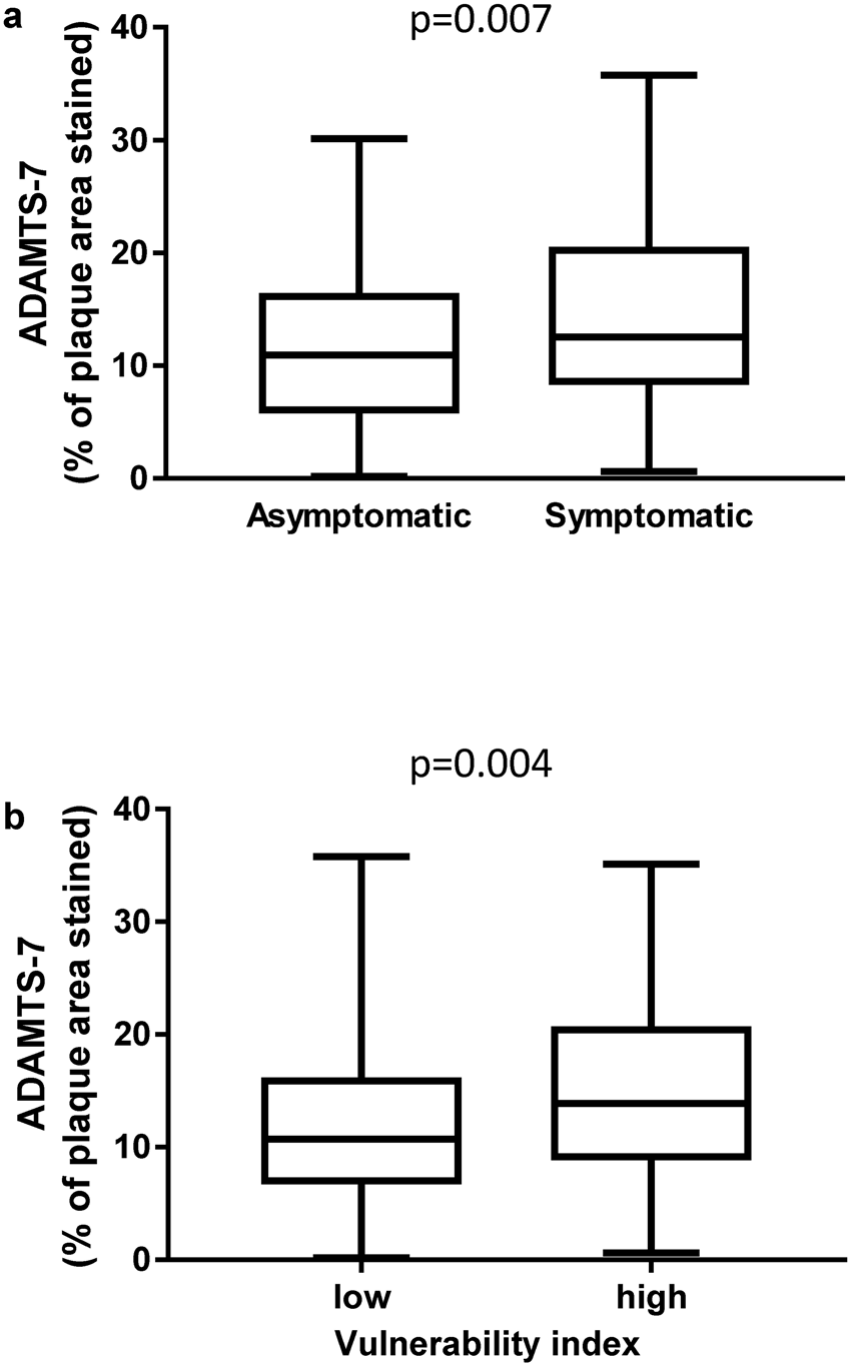
ADAMTS-7 is increased in symptomatic and vulnerable lesions. ADAMTS-7 (% of plaque area stained) is increased in carotid lesions removed by endarterectomy from symptomatic patients compared to lesions from asymptomatic patients (**a**). ADAMTS-7 is increased in lesions with a high vulnerability index (above median) (**b**). Vulnerability index was calculated based on lipid-, CD68-, hemorrhage-, SMC-, and collagen-staining (% of plaque areas stained). Values are presented as boxplots. Number of plaques = 206; Mann-Whitney test.

To investigate if ADAMTS-7 is associated with a stable or unstable plaque phenotype, expression levels of ADAMTS-7 were analyzed in 206 carotid human lesions using immunohistochemistry. Clinical characteristics of the patients are shown in Table 1. First, we compared the level of ADAMTS-7 staining in lesions from patients with cerebrovascular symptoms (transient ischemic attack, stroke, or amaurosis fugax) with patients without symptoms. ADAMTS-7 levels were increased in lesions from symptomatic patients compared with lesions from asymptomatic patients (Fig. 1a). In the next step, we investigated if ADAMTS-7 levels are associated with a more vulnerable plaque phenotype. ADAMTS-7 levels were correlated with plaque components known to be important for plaque stability. A positive correlation was found between ADAMTS-7 and CD68-staining, whereas a negative correlation was found to SMCs (Table 2). In addition, ADAMTS-7 correlated inversely with the major extracellular matrix plaque proteins collagen and elastin, whereas it correlated positively with the lipid content of the plaques. Using a vulnerability index of the plaques^15^, including lipid, macrophage, hemorrhage, SMC, and collagen content, revealed that ADAMTS-7 was increased in more vulnerable plaques (Fig. 1b). Moreover, ADAMTS-7 displayed a negative correlation to MMP-2, which has been associated with a more stable plaque phenotype^16^, as well as to MMP-3, whereas a positive association was seen to MMP-9 (Table 2). ADAMTS-7 also displayed a trend towards a positive association to MMP-1 (Table 2). ADAMTS-7 showed no significant association to MMP-10 or TIMP-2, but was positively associated with TIMP-1 (Table 2). Previous studies have shown that ADAMTS-7 expression is up-regulated in cultured macrophages and SMCs following stimulation with TNF-α^7, 11^. In agreement with this, ADAMTS-7 correlated positively with plaque amounts of TNF-α (r = 0.15, p = 0.04) in the present study (Table 2). Taken together, these results indicate that increased levels of ADAMTS-7 are associated with an unstable plaque phenotype.

**Table 1:** Clinical characteristics of the patient cohort.

	Total (n = 201)	Asymptomatic (n = 95)	Symptomatic (n = 106)	p
Age, years	69.1 (SD 8.3)	67.1 (SD 6.7)	71.0 (SD 9.2)	0.0005
Body mass index (kg/m^2^)	26.8 (SD 4.1)	27.0 (SD 4.0)	26.6 (SD 4.2)	0.5
Gender	136 male/65 female	65 male/30 female	71 male/35 female	0.8
Degree of stenosis, %	90 (IQR 80–95)	90 (IQR 85–95)	85 (IQR 75–95)	0.01
Diabetes mellitus, %	34 (n = 68)	24 (n = 23)	42 (n = 45)	0.006
Smoking, %	34 (n = 68)	40 (n = 38)	28 (n = 30)	0.08
Fasting lipoproteins, mmol/L				
Total cholesterol	4.3 (SD 1.1)	4.3 (SD 1.1)	4.4 (SD 1.2)	0.7
LDL cholesterol	2.6 (SD 1.0)	2.5 (SD 0.9)	2.6 (SD 1.1)	0.4
HDL cholesterol	1.1 (IQR 0.9–1.3)	1.1 (IQR 0.9–1.4)	1.1 (IQR 0.9–1.2)	0.9
Triglycerides	1.3 (IQR 0.9–1.8)	1.3 (IQR 0.9–1.8)	1.2 (IQR 1.0–1.8)	0.5
Hemoglobin, g/L	141(SD 13)	142 (SD 13)	139 (SD 13)	0.1
CRP	3.9 (IQR 2.0–6.4)	3.8 (IQR 1.8–6.0)	4.0 (IQR 2.0–7.0)	0.3
White blood cell count, 10^9^/L	7.9 (SD 2.0)	7.9 (SD 1.9)	7.9 (SD 2.0)	1.0
Statins, %	88 (n = 176)	92 (n = 87)	84 (n = 89)	0.1
Anti-hypertensive treatment, (%)	81 (n = 162)	83 (79)	78 (83)	0.4

Values are presented as mean and SD, or when not normally distributed as median with interquartile range (IQR). p represents the significance comparing the symptomatic and asymptomatic patient groups. CRP, C-reactive protein.

**Table 2:** Correlation of ADAMTS-7 (% of plaque area stained) in lesions to extracellular matrix, lipids, cells and MMPs.

	ADAMTS-7	
	r	p
*Extracellular matrix*		
Collagen^1^	−0.20	0.005
Elastin^1^	−0.18	0.009
*Lipids*		
Oil red O^2^	0.27	0.00007
*Cells*		
SMCs^2^	−0.17	0.017
CD68^2^	0.16	0.019
*MMPs and TIMPs*		
MMP-1^1^	0.13	0.08
MMP-2^1^	−0.19	0.008
MMP-3^1^	−0.22	0.002
MMP-9^1^	0.16	0.003
MMP-10^1^	0.07	0.33
TIMP-1^1^	0.28	0.00008
TIMP-2^1^	−0.024	0.7
*Cytokine*		
TNFα^1^	0.15	0.04

^1^Weight per gram plaque tissue. ^2^Percentage of plaque area stained.

### ADAMTS-7 localization in advanced atherosclerotic lesions

**Figure 2:**
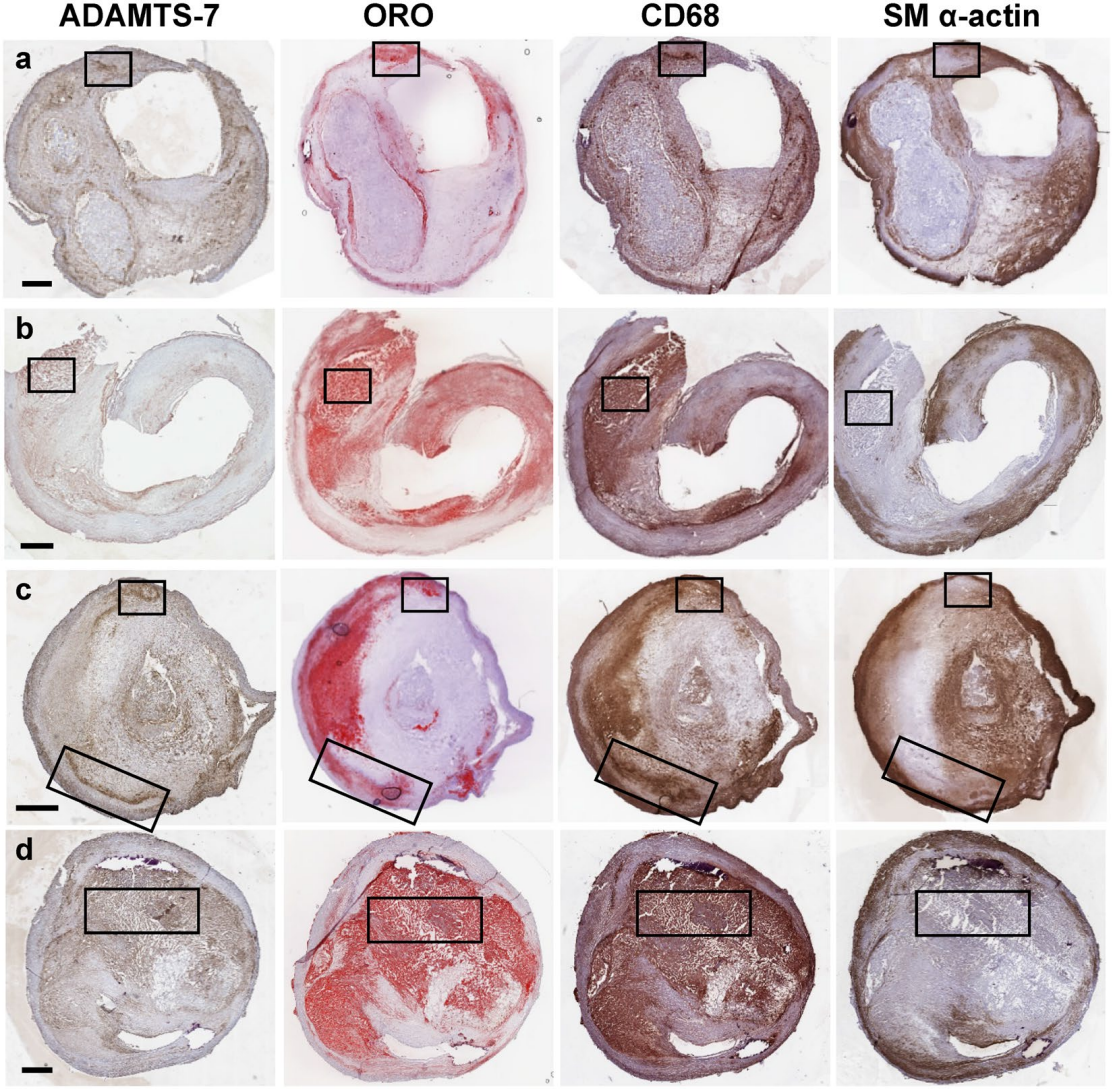
ADAMTS-7 is localized to areas staining positive for CD68 and lipids in human advanced carotid atherosclerotic plaques. Representative images are shown for staining of ADAMTS-7, lipids (Oil Red O, ORO), CD68, SMCs (SM α–actin) in four different atherosclerotic plaques (**a–d**). Examples of regions positive for ADAMTS-7, lipids, and CD68-staining are boxed. Scale bar: 1000 μm.

**Figure 3:**
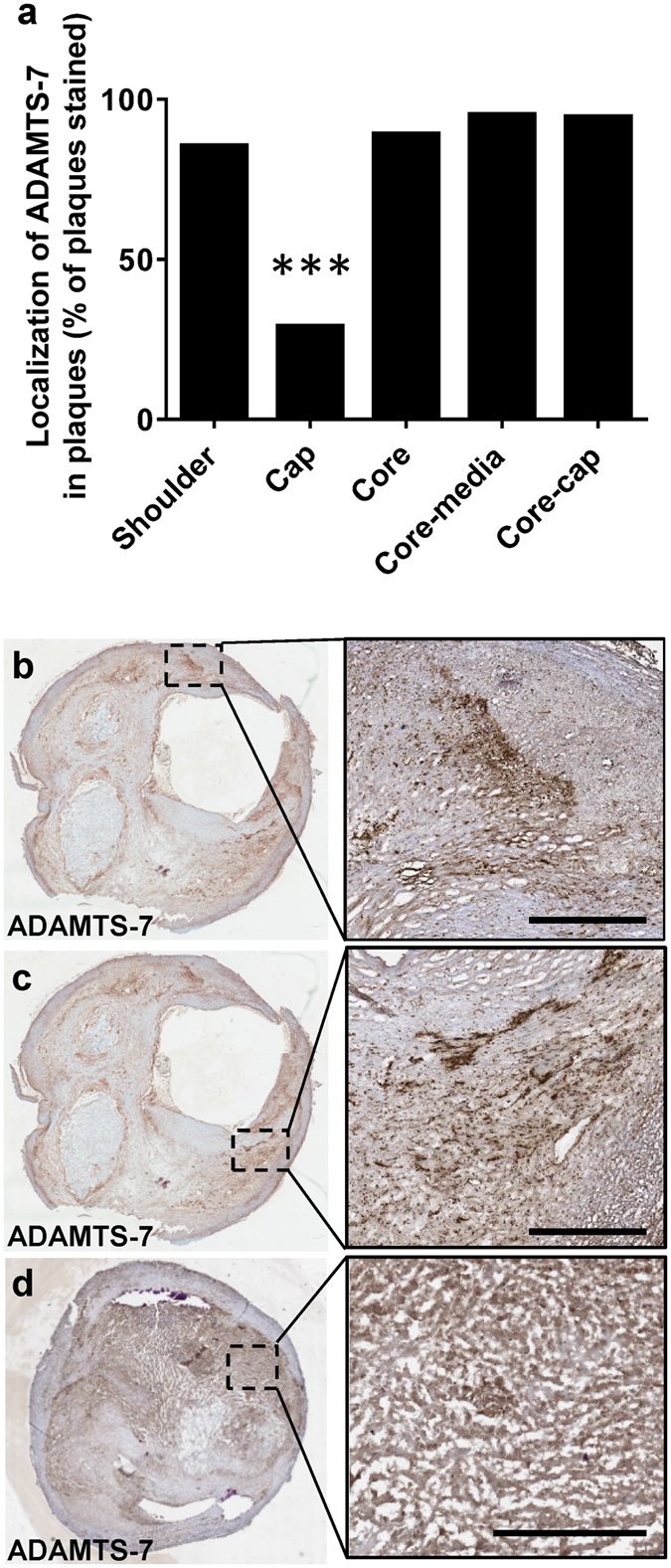
Localization of ADAMTS-7 in human carotid plaques. The presence of ADAMTS-7 (% of plaques stained) in different regions of the plaque were analysed (**a**). Representative images are shown for staining of ADAMTS-7 in shoulder regions (**b–c**), and in the core (**d**) of the plaques. Scale bars: 500 μm. p < 0.0001 (cap region compared to all of the other regions); Chi-square test.

**Figure 4:**
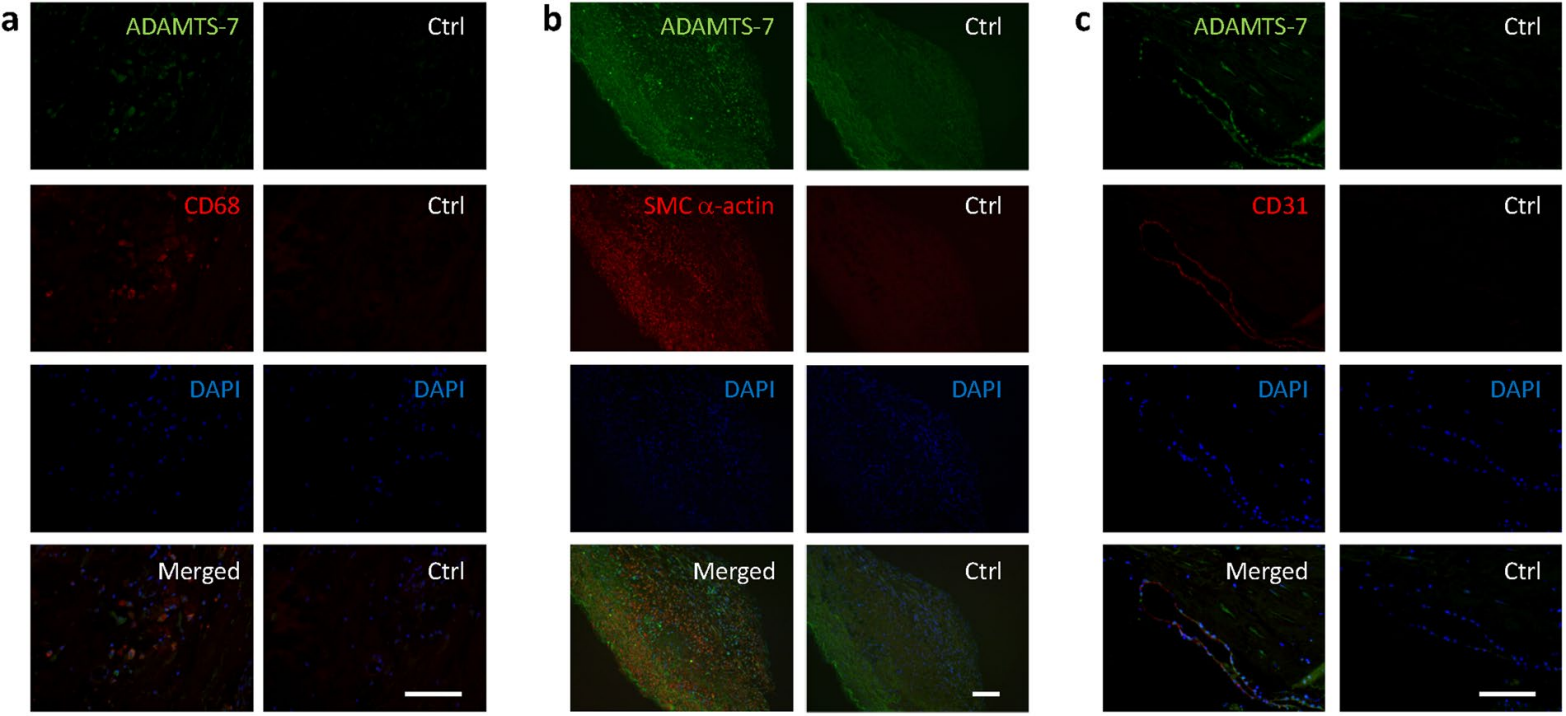
ADAMTS-7 co-stainings with CD68 (**a**), SMC α-actin (**b**), and CD31 (**c**). Human carotid plaque stained for ADAMTS-7 (green) and CD68 (**a**), SMC a-actin (**b**) or CD31 (**c**) (red), DAPI (blue) and merged together. Control sections are stained with isotype controls, DAPI, and merged. Scale bars: 10 µm.

To investigate the regional localization of ADAMTS-7 in human atherosclerotic lesions, we analyzed sections immunostained for ADAMTS-7. All lesions in the study were advanced, with high-degree of stenosis, large lipid-rich core areas, and CD68-stained areas. The analysis revealed positive immunostaining of ADAMTS-7 in all lesions investigated. ADAMTS-7 was mainly present in regions staining positive for CD68 and lipids (Fig. 2), although ADAMTS-7 staining was less widespread than the other stainings. An analysis of all plaques showed that ADAMTS-7 colocalized with CD68-staining in 207 of 208 plaques (99.6%) and with SMC α-actin in 157 of 208 plaques (76.4%). In addition, we performed a CD11b staining and compared with CD68- and ADAMTS-7-stainings (Supplementary Fig. S1). CD68 staining was more widespread than CD11b staining, which indicate that some of the CD68 positive cells may not be macrophages. ADAMTS-7 was present in CD11b positive regions, but also to some extent in other parts of the lesion. ADAMTS-7 was foremost localized to shoulder regions, to the cores, and to the borders between the media and the lipid-rich core and between the core and the cap, whereas it was less frequently observed in the cap region (Fig. 3). A co-staining of ADAMTS-7 and CD68 revealed cells positive for both markers (Fig. 4a). In addition, ADAMTS-7 colocalized to SMC α-actin in some regions (Fig. 4b), whereas in other regions no apparent colocalization was present (Supplementary Fig. S2). A colocalization of ADAMTS-7 and the endothelial cell marker CD31 was present in the lesions (Fig. 4c).

### Association of ADAMTS-7 to CV risk factors

The amount of ADAMTS-7 in lesions did not correlate with age, but was higher in men than in women (12.7% of plaque area stained (8.4–20.1) versus 10.1% (7.0–15.7), p = 0.03). There were no significant differences in ADAMTS-7 levels in lesions from diabetics (12.5% (8.3–21.2)) versus non-diabetics (11.6% (7.6–16.7) (p = 0.092), statin-treated (12.1% (7.9–18.3)) versus not statin-treated (10.3% (7.2–16.6)) (p = 0.55) or anti-hypertensive treated patients (12.0% (7.5–18.4)) versus not anti-hypertensive-treated (12.0% (8.8–17.8)) (p = 0.72). There were no associations between ADAMTS-7 and body mass index, cholesterol, triglycerides, LDL, HDL, CRP, white blood cell count, or HbA1c in blood (Supplementary Table S1). ADAMTS-7 was lower in current smokers versus non- and ex-smokers (10.0% of plaque area stained (6.9–13.8) versus 13.4% (8.1–19.9), p = 0.009). ADAMTS-7 levels did not correlate with the number of days between symptoms and removal of the plaque (r = 0.006, p = 0.95).

### ADAMTS-7 is associated with increased risk for postoperative CV events

**Figure 5:**
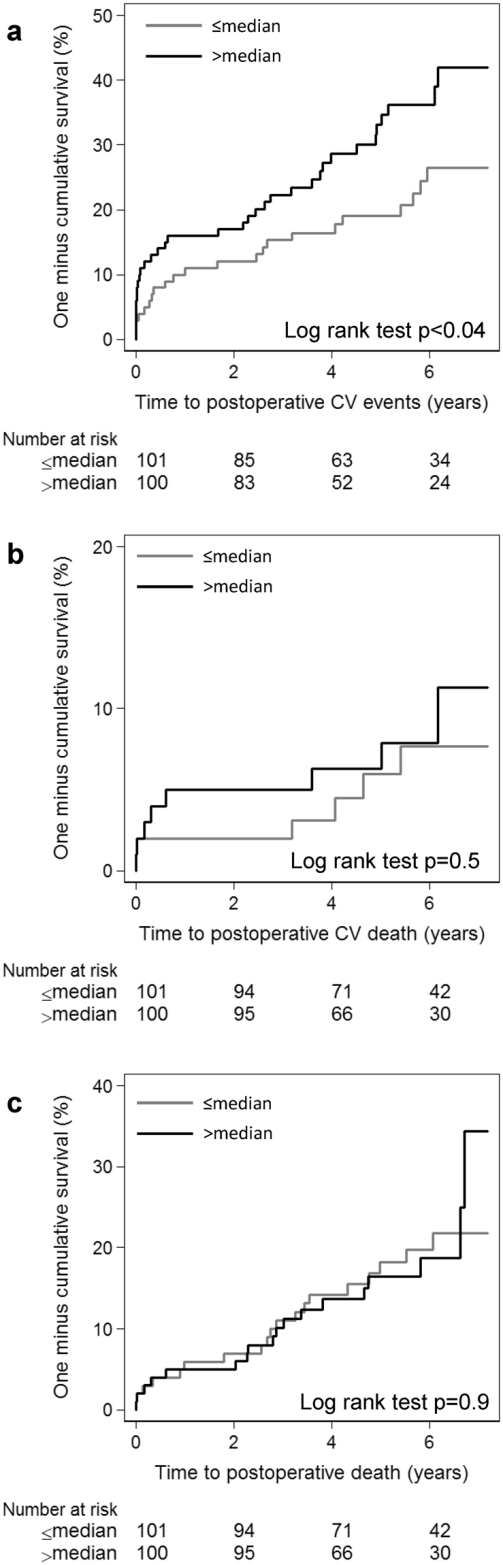
Association between ADAMTS-7 (% of plaque area stained) in lesions and risk of a future CV event. Kaplan-Meier curves illustrating risk for postoperative CV events (**a**), CV deaths (**b**) and all causes deaths (**c**) across ADAMTS-7 above or below the median.

To assess whether ADAMTS-7 levels in lesions is related to an increased risk of a future CV event or death, we compared ADAMTS-7 in patients with (n = 56) and without (n = 145) postoperative CV events (acute myocardial infarction, stroke, transient ischemic attack, amaurosis fugax, or CV-death; median follow-up time 57 (IQR 32-73) months). Comparing ADAMTS-7 levels above or below the median an association between high levels of ADAMTS-7 and an increased risk of a future CV event was found (Log rank test p = 0.04, Fig. 5). The Hazard-ratio for ADAMTS-7 above the median for a future CV event was 1.76 (95% confidence interval, 1.02–3.06; p = 0.04) in a Cox proportional hazard model after adjusting for potential confounders (sex and current smoking). Since ADAMTS-7 is increased in symptomatic lesions, we added symptoms to the model to address whether it is ADAMTS-7 or the presence of symptoms that is the actual predictor for postoperative events. Hazard-ratio for ADAMTS-7 above median for a future CV events was 1.71 (95% confidence interval, 0.98–2.97, p = 0.057) after adjusting for sex, current smoking and symptoms. We also analyzed asymptomatic and symptomatic patients separately. Interestingly, we found that high levels of ADAMTS-7 increased the risk for future CV in symptomatic patients (Log rank test p = 0.009), but not in asymptomatic patients (Log rank test p = 0.68). Hazard ratios for ADAMTS-7 above median for CV event were 2.63 (95% confidence interval, 1.24–5.57; p = 0.01) in symptomatic patients and 0.91 (95% confidence interval, 0.36–2.29; p = 0.84) in asymptomatic patients after adjusting sex and current smoking. These data show that ADAMTS-7 is an independent risk factor for postoperative events in symptomatic patients, but not in asymptomatic patients. No associations between ADAMTS-7 levels and postoperative CV death (n = 14 of 201 patients) or all causes mortality (n = 37 of 201 patients) were found (Fig. 5).

### A common genetic variant in the ADAMTS-7 locus is associated with CV death

**Figure 6:**
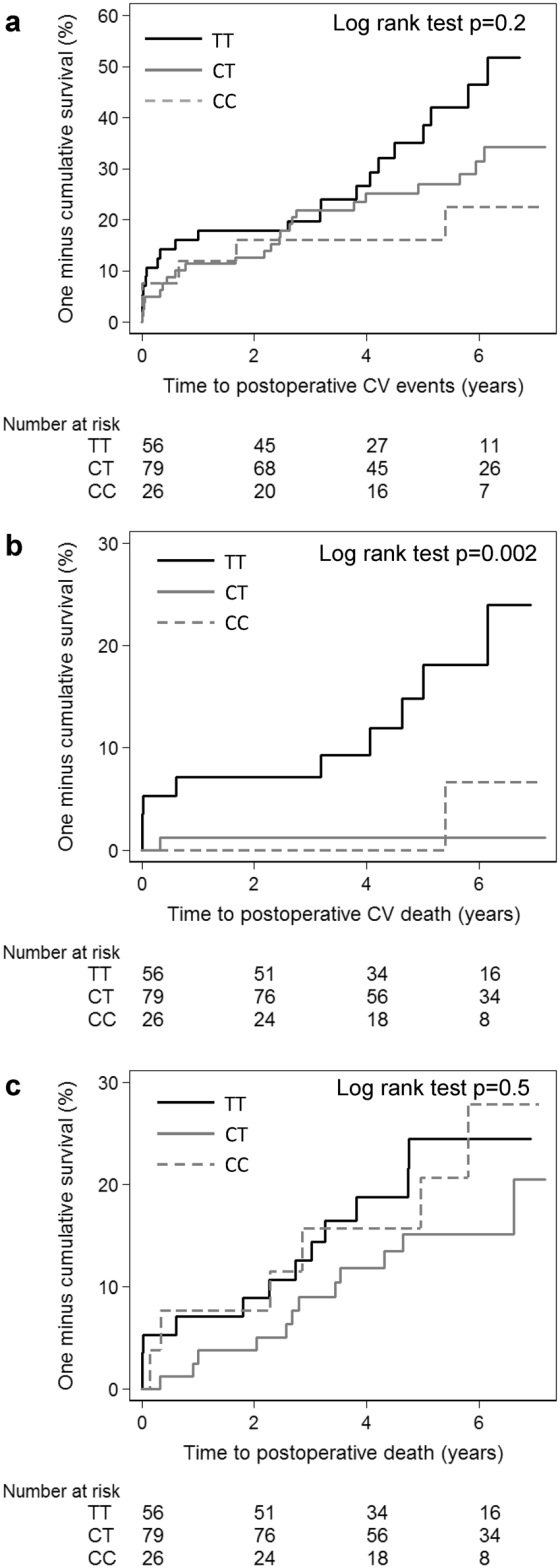
SNP rs7177699 in the ADAMTS-7 locus is associated with increased risk for a future CV death. Kaplan-Meier curves illustrating risk for postoperative CV events (**a**), CV deaths (**b**) and all causes deaths (**c**) across ADAMTS-7 genotypes in SNP rs7177699.

In the next step, we analyzed a genetic variant in the ADAMTS-7 locus and its association to a postoperative CV event or death. The SNP rs7177699, which is in strong linkage disequilibrium (R^2^ = 0.967) with rs3825807, previously identified as a susceptibility gene variant for CAD and acute myocardial infarction in a GWAS study,^3^ was analyzed. Rs7177699 was significantly associated with CV deaths, but not with all causes mortality or postoperative CV events in our cohort (Fig. 6). The frequency of the T allele was 0.598 in rs7177699 and the hazard-ratio for the TT genotype versus the CT and CC genotype in rs7177699 for future CV death was 10.17 (95% confidence interval, 2.19–47.28; p = 0.003) in the Cox proportional hazard model after adjusting for age and sex. SNP rs7177699 did not associate with ADAMTS-7 levels in the lesions (p = 0.86, Kruskal-Wallis test, Supplementary Fig. S3).

## Discussion

In the present study, ADAMTS-7 content was determined in a large sample of human plaques well characterized histologically and obtained from a carefully followed up patient cohort for future CV events. Our data show that ADAMTS-7 in human atherosclerotic lesions is associated with a more vulnerable plaque phenotype (Fig. 1b), being positively associated with CD68-staining and lipids, but negatively associated with SMCs and collagen (Table 2). Furthermore, the finding of increased levels of ADAMTS-7 in lesions from symptomatic patients (Fig. 1a) and in patients with postoperative CV events (Fig. 5a) supports the notion that ADAMTS-7 is associated with plaque vulnerability. Being a matrix degrading protease^4^ ADAMTS-7 may play a role in plaque vulnerability by degradation of extracellular substrates. Additionally ADAMTS-7 may affect plaque stability via its stimulatory effect on the expression of inflammatory cytokines and proteases. Recently, a positive feed-back loop between ADAMTS-7 and TNF-α has been demonstrated in cartilage explants^5^. Stimulation of cartilage with ADAMTS-7 resulted in an induction of MMP -1, -9, -13, and -14 expression, whereas expression of MMP-3 was unaffected. It was suggested that ADAMTS-7 induces expression of TNF-α, which in turn leads to increased MMP expression resulting in degradation of collagen^5^. In agreement with this, the present study demonstrates a positive association between ADAMTS-7 levels in the lesions and TNF-α, MMP-1, and MMP-9, whereas a negative association was found to MMP-2 and MMP-3 (Table 2). MMP-1^17–19^ and MMP-9^16, 17^ expression is localized to shoulder regions of human atherosclerotic plaques and associated with plaque rupture. Overexpression of active MMP-9 or local adenovirus expression of pro-MMP-9 results in plaque disruption in atherosclerotic mice^20^. Deficiency of MMP-3 in atherosclerotic mice results in divergent results, *i.e*. more stable plaques in the aorta^21^ and less stable plaques in the brachiocephalic artery^22^ likely reflecting the different role of MMP-3 in SMCs and macrophages. On the other hand, MMP-2 is increased in fibrous carotid lesions^16^, and lesions from MMP-2 deficient mice displayed fewer SMCs^23^, consistent with a role of MMP-2 in SMC migration and increased plaque stability. Thus, being part of an inflammatory feedback loop, ADAMTS-7 may exert its effect on plaque vulnerability by promoting inflammation and matrix degradation via induction of MMPs and other metalloproteinases belonging to the ADAMTS family.

Investigation of ADAMTS-7 localization in advanced human carotid atherosclerotic plaques in the current study revealed that lipid- and CD68-rich areas (Fig. 2), including shoulder regions and the core of the plaque (Fig. 3), show the highest staining intensity. However, co-staining of ADAMTS-7 and SMC α-actin also revealed colocalization with SMCs in some regions (Fig. 4b), but not in others (Supplementary Fig. S2). In a recent study by Bauer *et al*. ADAMTS-7 was found to colocalize with SM α-actin positive cells in the media of coronary arteries and in the intima of fibrotic lesions^8^. Bauer *et al*.*’s* study is in line with results from ADAMTS-7-deficient mice, which display significantly reduced neointimal area upon wire injury of carotid or femoral artery^8, 9^. It is possible that ADAMTS-7, induced by an inflammatory stimuli, can be expressed by both SMCs and macrophages, and the impact or expression pattern of ADAMTS-7 is dependent on the cellular contents of the plaques. Thus, in neointimal thickenings or in fibrous lesions ADAMTS-7 may be expressed by SMCs affecting SMC migration, whereas in advanced atherosclerotic lesions with high content of lipid and inflammatory cells ADAMTS-7 is mainly associated with macrophages. This is supported by the fact that ADAMTS-7 is expressed in several tissues including musculoskeletal tissues, heart, brain, placenta, pancreas, liver ovary, kidney, testicle, lung and thymus^24, 25^, and in several of these tissues its expression is increased upon inflammatory stimuli. For example, THP-1 monocytes show increased ADAMTS-7 expression after stimulation with interferon-γ or TNF-α^11^ and cartilage explants increase ADAMTS-7 expression after TNF-α or interleukin-1β stimulation^26^. Furthermore, angiotensin II-treatment of mice associated with inflammatory kidney damage induces renal ADAMTS-7 expression^27^ and ADAMTS-7 is elevated in collagen-induced arthritic mice^6^. It is also possible that some of the CD68 stained cells in the analyzed carotid plaques in our study are in fact SMC-derived cells displaying macrophage markers. A recent report shows that a large percentage of cells expressing macrophage markers in human atherosclerotic plaques may be SMC-derived foam cells^28^. These findings are supported by studies in mice showing that 80% of SMC-derived cells in advanced plaques of apoE^−/−^ mice were not detected by traditional markers for SMCs, but instead displayed macrophage or mesenchymal stem cell markers^29^. Thus, it may be that in early or fibrotic lesions with less foam cells ADAMTS-7 is expressed by SMCs, but in advanced lesions some of these ADAMTS-7 expressing SMCs have become CD68 positive foam cells.

In the present study we found that SNP rs7177699 at the ADAMTS-7 locus was associated with postoperative CV death (Fig. 6). Our results are in agreement with previous GWAS studies showing strong associations of SNPs at the ADAMTS-7 locus and CAD^1–3^, although a comparison of patients with and without myocardial infarction and polymorphism in rs1994016 at the ADAMTS-7 locus was negative^2^. Rs7177699 is in strong linkage disequilibrium with the previously CAD associated SNP rs3825807^3^. We did not found any association between ADAMTS-7 amounts in lesions and rs7177699 (Supplementary Fig. S3), which is in agreement with a study by Pu *et al*. showing that ADAMTS-7 content in coronary atherosclerotic plaques from 44 individuals did not differ between genotypes of rs3825807^10^. They also demonstrated that the CAD associated allele in SNP rs3825807, leading to an amino acid substitution in the prodomain of ADAMTS-7, results in increased ADAMTS-7 maturation and activity, whereas no difference in the amount of the full-length protein was found^10^.

Smoking is associated with increased oxidation and inflammation. Moreover, tobacco smoke induces the release of microvesicles with proteolytic activity in macrophages^30^. Unexpectedly, we found that smokers had lower levels of ADAMTS-7 in the lesions compared to non-smokers (never smoked or ex-smokers). In the present study, we measured ADAMTS-7 content in the most stenotic part of the lesion, and it is possible that the effect of smoking on plaque components varies in different parts of the lesions.

A number of limitations of the present study should be noted: First, since the study it is not mechanistic, one cannot draw functional conclusions about ADAMTS-7 in human lesions. Second, the correlations of ADAMTS-7 to some of the MMPs and TNFα were weak. Third, the analysis was performed on a cross-section of the most stenotic part of the lesions. Since, the lesions can be heterogeneous, we cannot exclude that ADAMTS-7 content is different in other parts of the lesion. However, the correlation of ADAMTS-7 to lipids, CD68 and SMC α-actin levels, measured in adjacent cross-sections of the lesions, argues for a correlation of ADAMTS-7 to a more vulnerable phenotype. Fourth, the number of events in the genetic analyses is low, and therefore the results should be interpreted with caution. Nevertheless, our results are in agreement with previous GWAS studies, associating SNPs in the ADAMTS-7 locus to CAD. The strengths of the study are: (1) the first assessment of ADAMTS-7 content in a large amount of human plaques, both from symptomatic and asymptomatic patients; (2) the very thorough characterization of the plaques histologically and biochemically, enabling us to study the association of ADAMTS-7 to the plaque phenotype of vulnerability; (3) the link to the patient cohort, who donated the plaques, carefully followed up with telephone interviews and national registries, allowing the connection between plaque ADAMTS-7 and prediction of future CV events.

In conclusion, we show that ADAMTS-7 in advanced human carotid plaques is associated with vulnerable plaque characteristics, increased symptoms, and increased risk of CV events. These findings support previous studies of a potential proatherogenic role of ADAMTS-7.

## Materials and Methods

### Patients and sample preparation

Human carotid plaques (n = 206) were collected from 201 individuals by endarterectomy. The indications for surgery were plaques associated with ipsilateral symptoms (transient ischemic attack, stroke, or amaurosis fugax) and stenosis >70% (n = 106), measured by duplex, or plaques not associated with symptoms but with stenosis >80% (n = 100). All plaques from symptomatic patients were removed within 32 days after symptoms. Informed consent was given by each patient. The study was approved by the Regional ethics committee in Lund (approval no 472/2005) and conducted in agreement with the Declaration of Helsinki. All patients were preoperatively assessed by a neurologist. After surgical removal, the carotid plaques were snap-frozen in liquid nitrogen and 1-mm thick fragments were cut from the most stenotic region of the plaque for subsequent immunohistochemical studies. Plaque homogenates were prepared for assessment of protein expression as previously described^31^.

### Immunohistochemistry

Transversal tissue sections (8 µm thick) from the plaque fragments were stained for ADAMTS-7, vascular SMCs (smooth muscle α-actin, SM α-actin), macrophages (CD68), and lipids. Three different antibodies were evaluated for ADAMTS-7 staining (ab28557 from Abcam, sc-163642 from SantaCruz Biotechnology, and Orb10042 from Biorbyt). The antibody ab28557 has previously been reported to stain positively for ADAMTS-7 in carotid atherosclerotic plaques^10^ and was used for further analysis. For staining of ADAMTS-7, SM α-actin, and CD68-staining, sections were fixed in acetone, permeabilized with 0.5% Triton-X, and incubated in phosphate-buffered saline (PBS) containing 3% H_2_O_2_ to neutralize endogenous peroxidase activity. After pre-incubation in PBS containing 10% serum, sections were incubated in either rabbit anti-ADAMTS7 antibody (ab28557, Abcam, Cambridge, UK), mouse anti-SM α-actin antibody (clone 1A4, DakoCytomation, Glostrup, Denmark), mouse anti-CD68 antibody (clone KP1, DakoCytomation), mouse anti-human glycophorin A/CD235a antibody or rabbit IgG isotype control antibody (Ab172730, Abcam) over night. After washing, sections were incubated with secondary biotinylated antibodies (goat anti-rabbit, Vector Laboratories or rabbit anti-mouse, DakoCytomation) diluted in PBS with 10% serum for 1 hr. Sections were washed and developed using DAB peroxidase substrate kit, according to manufacturer’s instructions (ImmPACT DAB, Vector Laboratories, Peterborough, UK). After color development the slides were immediately washed under tap water. Counterstaining was achieved using Mayer’s hematoxylin. CD11b-staining (ab52478, rabbit monoclonal anti-CD11b, Abcam) was performed as described above, but blocked with 10% bovine serum albumin, and detected with MACH 3Rabbit Probe followed by MACH 3Rabbit HRP-Polymer (Histolab). Slides were mounted with VectaMount (Vector Laboratories) and observed under a light microscope. Specificity of staining was confirmed by absence of staining in sections incubated with IgG isotype control antibody or exclusion of primary antibodies. Lipid content was determined by Oil red O staining and collagen content by Masson trichrome staining as previously described^32^. Areas of the different stainings in the plaques were quantified blindly using Biopix iQ 2.1.8 (Gothenburg, Sweden) after scanning with Scansope Console Version 8.2 (LRI imaging AB, Vista California, USA) and photographed with Aperio image scope v.8.0 (Aperio, Visat California, USA). Areas of the different stainings were normalized to the total plaque area and expressed as % of plaque area stained. A histological vulnerability index of the plaques were determined as the sum of CD68-, lipid (Oil red O)- and hemorrhage (glycophorin A)-stainings divided by the sum of SMC- (SM a-actin)- and collagen (Masson trichrome)-stainings (each staining was measured as % of plaque area stained)^15, 33, 34^. The vulnerability index of the plaques was divided in above and below median representing high and low vulnerability, respectively.

### Immunofluorescence

Immunofluorescence stainings were performed on 4% formalin-fixed paraffin-sections, which were deparaffinized and pretreated by boiling 15 min in 10 mM sodium-citrate buffer pH 6.0, 0.05% Tween-20 for antigen retrieval. Sections were permeabilized in 0.5% Triton-X in Tris-buffered saline (TBS) and incubated in 0.3% H_2_O_2_ in methanol for 30 min to neutralize endogenous peroxidase activity. After blocking with 10% BSA in TBS, rabbit anti-ADAMTS7 antibody (ab28557, Abcam, Cambridge, UK) together with either mouse anti-CD68 (clone KP1, DakoCytomation), mouse anti-CD31 (M0823, DakoCytomation), or mouse anti-smooth muscle cell actin (M0851, DakoCytomation) were added, followed by goat anti-rabbit Alexa 488 (ab150077, Abcam, Cambridge, UK) and donkey anti-mouse Alexa 555 (ab150106, Abcam, Cambridge, UK) diluted in TBS with 1% BSA. Sections were then incubated with 0.03% Sudan black B (ICN Biomedicals) in 70% ethanol, before mounting in Vectashield Mounting Medium with DAPI (Vector Laboratories). Specificity of staining was confirmed by IgG isotype control antibodies.

*Assessment of collagen, elastin, MMPs, TIMPs, and TNF-α in plaque homogenates* Collagen was measured using Sircol soluble Collagen assay (Biocolor, Carrickfergus, Northern Ireland, UK) and elastin was measured using Fastin Elastin assay (Biocolor, Carrickfergus, Northern Ireland, UK). Matrix metalloproteinase (MMP)-1, -2, -3, -9, -10 were analyzed with Mesoscale human MMP ultrasensitive kit (Mesoscale, Gaithersburg, MD, USA) and tissue inhibitors of metalloproteinases (TIMPs; -1, -2) and TNF-α were analyzed with Luminex technology (Millipore Corporation, MA, USA). All analyses were normalized against wet weight of the plaque.

### Clinical follow up

The Swedish national inpatient health register was analyzed in order to identify postoperative CV events, with corresponding International Classification of Diseases, Tenth Revision (ICD-10) codes G45 and G46, I20 to I25 from October 2005 to December 2012. This is a nation-wide validated register where more than 99 percent of all somatic (including surgery) and psychiatric hospital discharges are registered^35^. In doubtful cases, information was gained through telephone interviews and medical chart reviews. All causes of death were confirmed through the National Population Register.

### Definition of outcomes

Each CV event was registered and analyzed separately. Events occurring in the first 24 hours postoperatively were considered as procedure-related and assumed as intraoperative for the analysis. Patients suffering more than one episode of the same event (for example, patients with multiple strokes) were classified as suffering multiple events. In these cases only the first chronological event was taken into account in the survival analysis.

### SNPs

DNA was extracted from whole blood from 161 patients and the rs7177699 was genotyped using the HumanOmniExpressBeadChip and iScan system (Illumina, San Diego, CA, USA).

### Statistics

Variables are presented as mean (standard deviation, (SD)), median (interquartile range (IQR)), or percentages. Two-groups comparisons of normally distributed data were analyzed by t-test and non-normally distributed variables were analyzed by Mann-Whitney test. Spearman rho was used to determine correlations. Categorical variables were analyzed by Pearson-Chi square test. When post-hoc tests were performed, nominal p-values were multiplied with the number of tests. Kaplan-Meier curves with Log rank test were done dividing patients with ADAMTS7 above or below median. Cox proportional hazard regression model was used to correct for confounders. The CV risk factors age, sex, smoking, cholesterol, anti-hypertensive treatment, and diabetes were included as potential confounders if they were significantly associated with ADAMTS-7 levels (p < 0.05). In the Cox proportional hazard regression model of CV death and ADAMTS-7 SNP, age and sex were included as potential confounders. IBM SPSS Statistics 22 or Stata statistical software 13 was used for the statistical analysis. Significance was considered at p < 0.05.

## Electronic supplementary material

Supplementary file
