# Supplementary material for: ADAMTS-7 is associated with a high-risk plaque phenotype in human atherosclerosis
Source: Sci Rep. 2017 Jun 16;7:3753. doi: 10.1038/s41598-017-03573-4 (PMC5473877; doi:10.1038/s41598-017-03573-4)
Supplement: Supplementary file 1 — Supplementary file [file 41598_2017_3573_MOESM1_ESM.pdf]

Supplementary information

ADAMTS-7 is associated with a high-risk plaque phenotype in human atherosclerosis

Eva Bengtsson, Karin Hultman, Pontus Dunér, Giuseppe Asciutto, Peter Almgren, Marju Orho-Melander, Olle Melander, Jan Nilsson, Anna Hultgårdh-Nilsson, and Isabel Gonçalves

Supplemenatary table S1

Supplementary figure S1-3

Supplementary Table S1

Correlation of ADAMTS-7 (% of plaque area stained) in lesions to cardiovascular risk factors.

	ADAMTS-7	
	r	p
Age, years	0.049	0.49
Body mass index (kg/m^2)	0.033	0.64
Degree of stenosis, %	0.063	0.37
Fasting lipoproteins, mmol/L		
Total cholesterol	-0.077	0.29
LDL cholesterol	0.017	0.82
HDL cholesterol	-0.14	0.063
Triglycerides	0.049	0.51
Hemoglobin, g/L	0.079	0.27
CRP	-0.136	0.065
White blood cell count, 10^9/L	-0.085	0.23

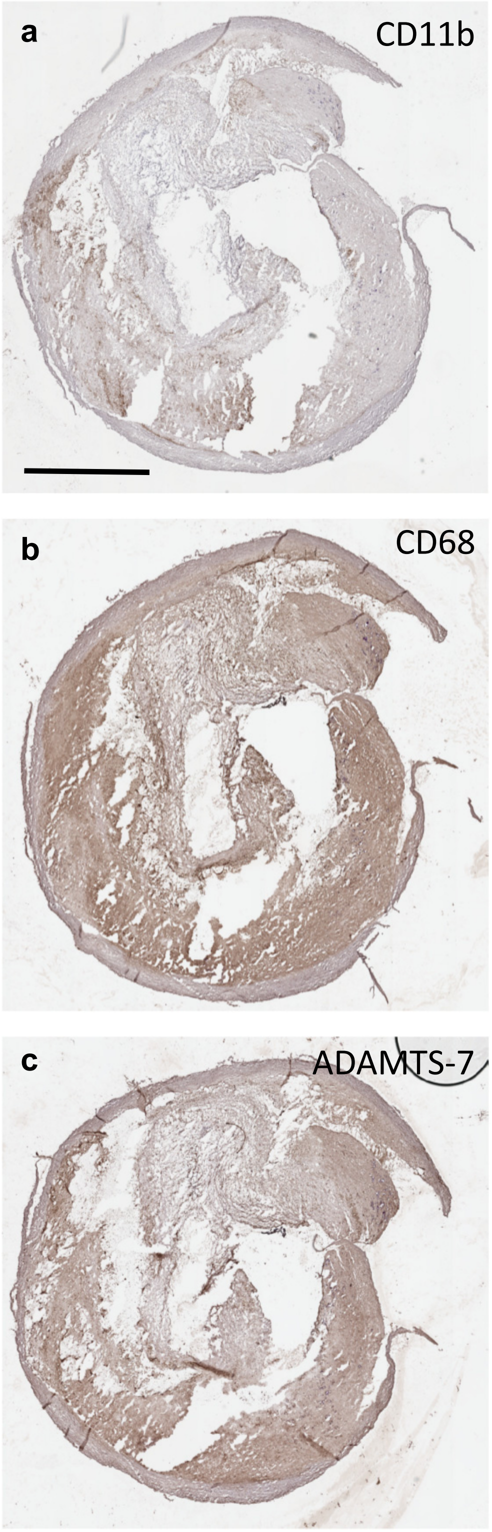

Supplementary Figure S1.
Human carotid plaque stained with anti-CD11b (**a**), anti-CD68 (**b**), and anti-ADAMTS-7 (**c**). Scalebar 2 mm.

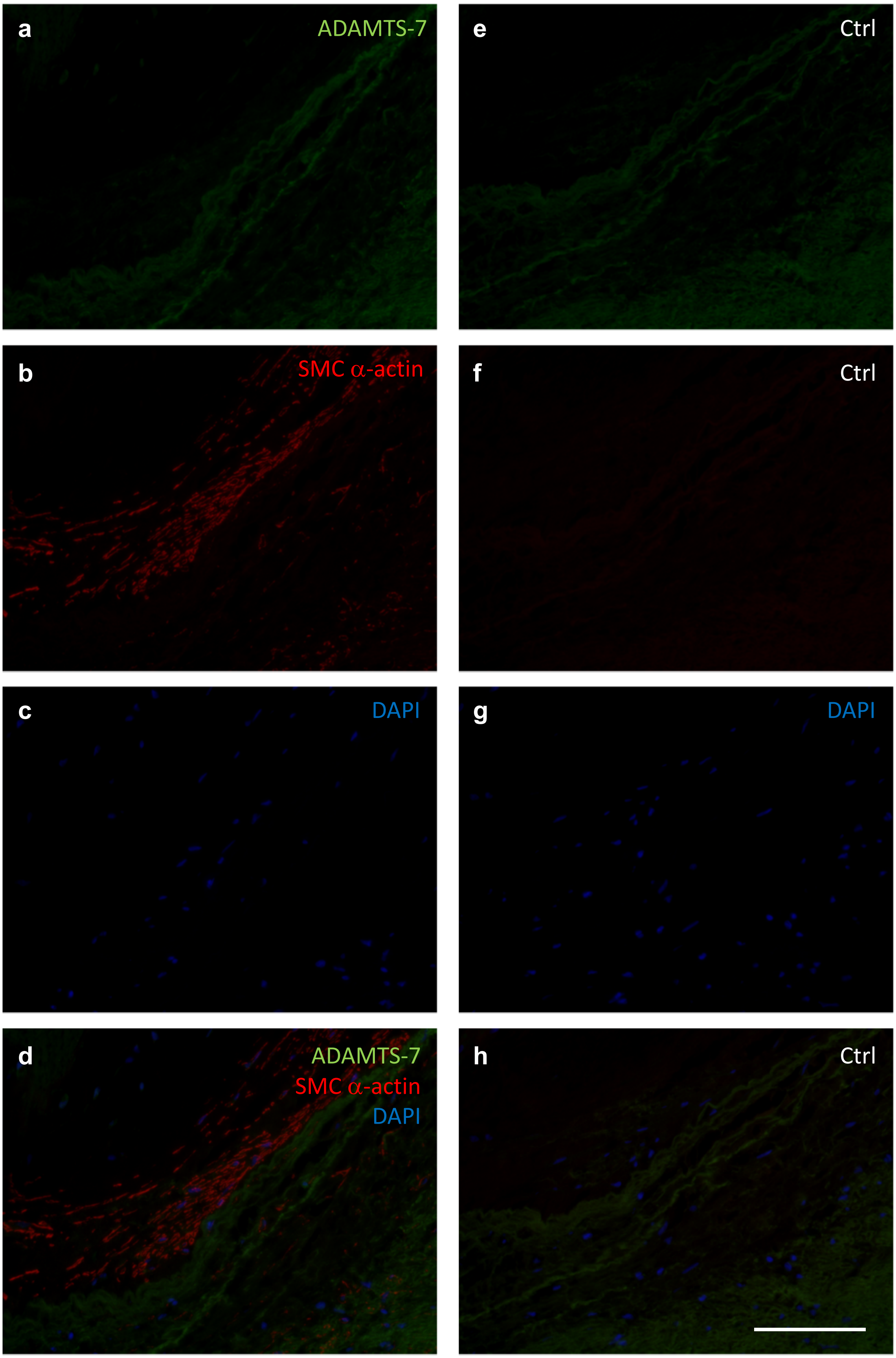

Supplementary Figure S2.
ADAMTS-7 and SMC a-actin co-staining. Human carotid plaque stained for ADAMTS-7 (green) (**a**), SMC a-actin (red) (**b**), DAPI (blue) (**c**) and merged together (**d**). Section stained with isotype controls, DAPI, and merged are shown in (**e-h**). Scale bar: 10 μm.

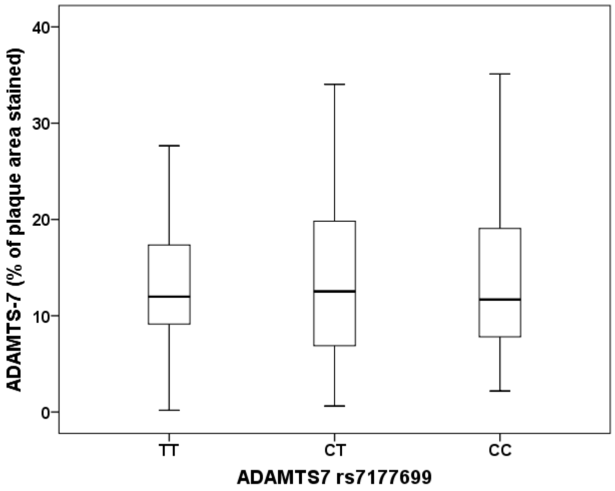

Supplementary Figure S3.
Genotypes of rs7177699 do not associate with ADAMTS-7 levels in lesions. The genotype of rs7177699 was determined in 161 patients. Values are presented as boxplots. p=0.86; Kruskal Wallis test.
